# Eyelid Eccrine Poroma: A Case Report and Literature Review

**DOI:** 10.7759/cureus.60316

**Published:** 2024-05-15

**Authors:** Silvia Pérez-Trigo, Regina-María López-Ladrón-García-Borbolla, Enrique Mencía-Gutiérrez, María Garrido-Ruíz, Álvaro Bengoa-González

**Affiliations:** 1 Ophthalmology, 12 de Octubre Hospital, Complutense University, Madrid, ESP; 2 Pathology, 12 de Octubre Hospital, Complutense University, Madrid, ESP

**Keywords:** sweat duct, adnexal neoplasm, benign, eccrine poroma, eyelid

## Abstract

Eccrine poroma is a rare benign adnexal tumor arising from intradermal cells of eccrine sweat ducts. At least two-thirds of eccrine poromas present on the extremities, most commonly on the palms and soles. They are scarcely found on the face; to date, only 11 cases of eyelid poromas have been reported in PubMed. Biopsy excision with a free margin is necessary to distinguish it from malignant lesions and avoid recurrence with possible transformation to porocarcinoma. We present the case of a 23-year-old male with a histopathological confirmation of poroma using staining with hematoxylin-eosin on the eyelid, previously clinically diagnosed with molluscum contagiosum. After four years, he has not experienced a recurrence.

## Introduction

Poroma is a benign adnexal neoplasm arising from intraepidermal cells of eccrine or apocrine sweat ducts, first described by Goldman et al. in 1956 [1]. More than two-thirds of poromas are located on the extremities, most commonly on the palms and soles of the feet, or on the sides of the foot [2]. Eyelid involvement in eccrine poromas is a rare occurrence. A search of the medical literature (PubMed) indicates 11 previous reports of eyelid eccrine poroma [3-13]. Poroid neoplasms include eccrine poroma, apocrine poroma, hidroacanthoma simplex, and dermal duct tumor [14]. Eccrine poromas are derived from cells of the other layer of acrosyringium and the upper dermal eccrine duct, both epidermal and dermal [1]. Poromas usually occur in middle-aged patients, with a male predominance [14]. Ultraviolet radiation does not seem to be a key pathognomonic factor in poroma development, nor a pathogenic factor, evolving primarily in the deep dermis which is less affected by ultraviolet radiation [15]. On the other hand, several correlations have been found between long-term radiation exposure and the development of poroma [16]. We describe the case of a 23-year-old male (the youngest described at the time of surgery), with a lesion on the upper eyelid of the right eye and aspect of molluscum contagiosum, and we analyze all cases of eyelid poromas published in the literature. Complete excisional biopsy with clear margins is the appropriate treatment to prevent potential malignancy to porocarcinoma and recurrence [15].

## Case presentation

**Figure 1:**
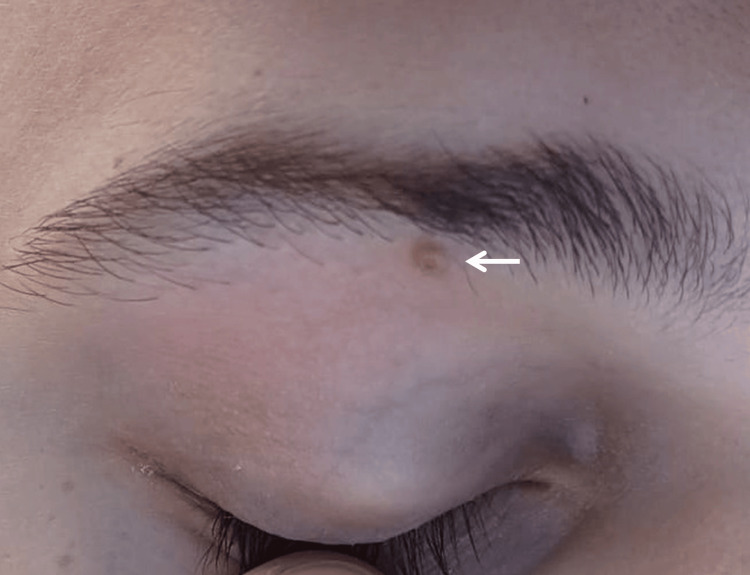
Clinical image of the lesion measuring 3 mm in diameter located in the right upper eyelid in the middle third into the crease and eyebrow just at the level of the first follicles. It is nodular, solid, and non-tender. The lesion is reddish-pink in color, unpigmented, and without telangiectatic vessels.

A 23-year-old male presented with a single asymptomatic skin eyelid lesion that had slowly grown over two years. It was located on the upper right eyelid, in the middle third, between the crease and eyebrow just at the level of the first follicles. It had a well-defined nodular-regular silhouette and was non-tender, solid, and umbilicated. It was reddish-pink in color, without additional pigmentation or telangiectatic vessels. It was scaly and not ulcerated with spontaneous occasional bleeding but without a specific bleeding point. The lesion measured 3 mm in diameter (Figure 1).

**Figure 2:**
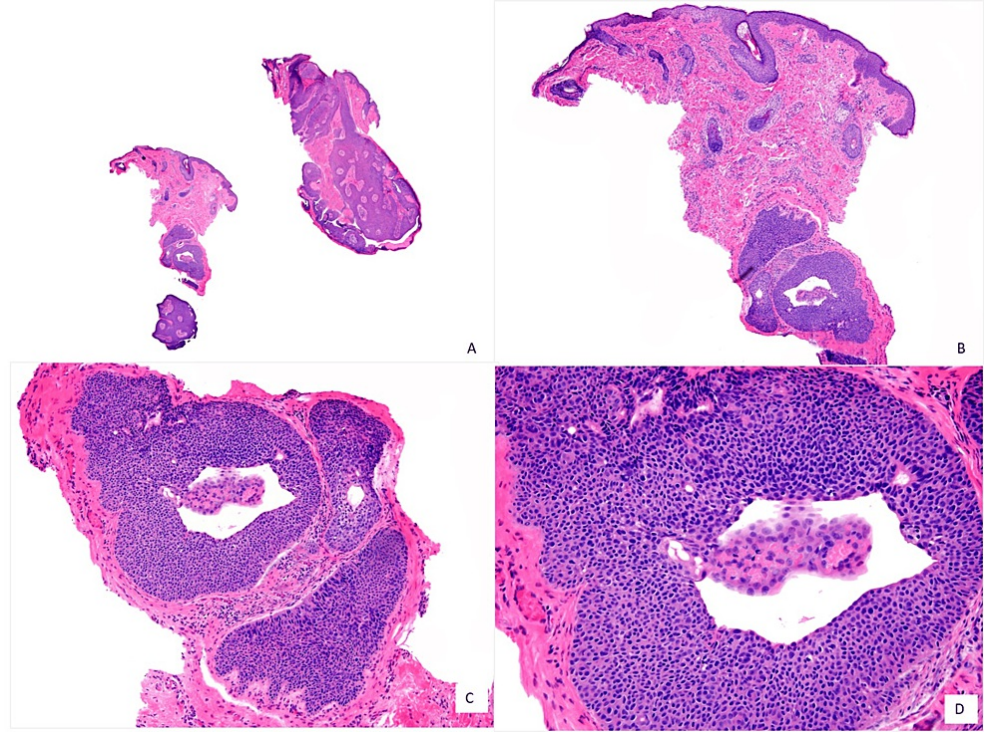
(A) Microscopic examination with the hematoxylin-eosin (H&E) stain of the resected specimen, in a panoramic view showing a well-defined nodular silhouette with the tumor completely excised (H&E, 4×). (B) The tumor is connected to the overlying epidermis and extends into the papillary and reticular epidermis. Circumscribed lobulated growth pattern is observed, with thicket cords of the tumor cells that surround a vascular fibrotic stroma (H&E, 10×). (C, D) The tumor is composed of neoplastic proliferation consisting of a mass of cubic monomorphic epithelial cells, originating in the epidermis toward the dermis showing the formation of well-defined lumens (H&E, 40×), covered by cuticle or intracytoplasmic lumens (H&E, 100×).

The clinical diagnosis was molluscum contagiosum. The patient had no other lesions on the skin and eyelids. The remainder of the ophthalmic examination was within normal limits. An excisional biopsy under local anesthesia was performed with macroscopic free margins on the eyelid skin (clearance of 2 mm). A microscopic examination with the hematoxylin-eosin stain of the resected specimen showed a well-defined nodular silhouette with the tumor completely excised in a panoramic view (Figure 2A). The tumor was connected to the overlying epidermis and extended into the papillary and reticular epidermis. A circumscribed lobulated growth pattern was observed, with thick cords of the tumor cells that surrounded a vascular fibrotic stroma (Figure 2B). The tumor was composed of a neoplastic proliferation consisting of a mass of cubic monomorphic epithelial cells, originating in the epidermis toward the dermis, showing the formation of well-defined lumens (Figure 2C), covered by cuticle or intracytoplasmic lumens (Figure 2D).

The diagnosis was an eccrine poroma. There has been no local recurrence in a follow-up of four years, nor the appearance of other skin or eyelid lesions. The eyelid aesthetic and functional results were very satisfactory.

## Discussion

Poroma is a benign cutaneous neoplasm related to the acrosyringium, and it is included in a group of benign adnexal neoplasms with ductal differentiation [1]. They appear preferentially on the palms or soles, and rarely in other areas, such as the face [14]. In primary skin lesions, sweat gland tumors account for approximately 1% of cases. Eccrine and apocrine poromas are believed to account for approximately 10% of these [15]. On the eyelids, barely 11 cases of poroma have been reported [3-13]. The biological potential in all different histological subtypes of poromas is the same, both eccrine and apocrine [9]. Our case, the 12th, is of eccrine type as it does not have apocrine differentiation, although the apocrine subtype in poromas has never been described with eyelid involvement. Sometimes, tumor cells can show a squamous appearance or be clear-pigmented. Poroma is usually non-pigmented (<20%) and it may be underdiagnosed due to confusion with other pigmented lesions [13]. Selected differential diagnoses must be made with benign sweat gland tumors such as hidradenoma, syringoma, and chondroid syringoma among others, as well as with irritated seborrheic keratosis. Differential diagnosis must also be made with a malignant eccrine sweat gland tumor as porocarcinoma, and with a basal cell carcinoma, squamous cell carcinoma, sebaceous gland carcinoma (which can be difficult to differentiate clinically), and, in general, with other lesions of the eyelid and periocular skin [9,13,17]. The exact etiology is unknown [18].

None of the described poromas had been correctly diagnosed preoperatively (Table 1). Most lesions were slowly growing asymptomatic ones, as in the present case. Two cases presented pain and itching and two cases had a bad smell [8,13]. In four (33%) cases the misdiagnosis was with carcinomas, in the other four cases, it was with benign lesions, and there is no data for the remaining four. The current case was diagnosed as molluscum contagiosum. The histopathological examination is crucial for its definitive diagnosis. Mitotic figures may be present and can be numerous in traumatized lesions [19], but no atypical mitotic figures were encountered in any case. It rarely progresses to porocarcinoma [17]. Therefore, an immunohistochemical study may be necessary to rule out malignancy in case of doubt with embryonic carcinogen antigen and for periodic acid-Schiff [18,19].

**Table 1:** Summary of cases of eccrine poroma involving the eyelids. ND = not determined; BCC = basal cell carcinoma; SCC = squamous cell carcinoma

Author/Year of publication/Reference number	Age (years)/ Sex	Eyelid/Location	Size (mm)	Form/Color	Follow-up (months) Previous/Post-surgery	Other skin lesions	Clinical diagnosis
Fujita et al. 1986 [3]	70/F	RC/Inner	10 × 10 × 9	Dome/Red	2/12	Siringocystadenoma papilliferum	Keratinous mass
Vu et al. 2001 [4]	71/M	RL/Inner	15 × 13	Nodule/Orange	96/36	BCC	BCC
Chen et al. 2006 [5]	76/M	RU/Lateral	3 × 3	Papule/Red	24/ND	ND	Fibroma
Rabady et al. 2008 [6]	71/F	RL/Lateral	3 × 5	Papule/Red	ND/12	Nevus	ND
Iwasaki et al. 2008 [7]	91/M	RL/Medial	4 × 5	Nodule/Red	12/ND	ND	BCC
Ahuja et al. 2019 [8]	70/M	RU/Medial	30 × 10	Nodule/Pigmented	72/6	Eccrine poroma	ND
Mencía-Gutiérrez et al. 2020 [9]	35/M	LU/Medial	6 × 3	Nodule/Skin color	12/36	No	BCC
Sharma et al. 2020 [10]	58/M	LU/Lateral	10 × 10	Nodule/Red-blue	ND/ND	ND	Capillary hemangioma
McCoskey et al. 2021 [11]	63/M	LL/Lateral	4 × 4	Papule/Red	3/ND	ND	ND
Kalamkar et al. 2021 [12]	70/M	RL/Medial	40	Large mass/Red-blue	180/ND	ND	BCC/SCC
Bogomolets et al. 2023 [13]	30/F	LL/Medial	12 × 14	Nodule/Pigmented	180/9	ND	Seborrheic keratosis
Current case	23/M	RU/Medial	3 × 3	Nodule/Red	24/24	No	Molluscum contagiosum

All cases collected in this study were of solitary presentation in the eyelid, being rarely multiple in other locations [18]. The average age was 66.66 years (range = 23-91 years); it was lower in females, at 57 years (range = 30-70 years), compared to males, at 61.88 years (range = 23-91 years). The proportion of involvement between females and males was 1:3. The patient described here was a 23-year-old male at the histopathologic diagnosis. Only two cases have been described previously under 40 years of age [9,13].

Regarding its eyelid location, eight were present on the right side (four, 50% in the middle part) and four on the left side (two in the middle part and two in the lateral part). There was no preference regarding localization on the upper eyelid or lower eyelid. It was more common on the right side in males (6/8), as in the current case, and on the left side in females (3/4). No previously reported case had affected the free edge of the eyelid. The average previous evolution time of all cases was 62.5 months (range = 2-180 months), that is to say, the pathological process was almost always slow [20]. Regarding its dimensions, the average size was almost 12 mm in diameter (range = 3-40 mm), making our case one of the smallest diagnosed with only 3 mm in diameter. Six (50%) cases were 5 mm or less in size. In most cases, its color was reddish (eight cases) or without special coloration (two cases), and two cases were pigmented [8,13]. No recurrence was observed with an average follow-up of 19.28 months (range = 6-36 months). In four (33%) cases, there were other associated lesions, including a preauricular basal cell carcinoma, multiple facial nevi, syringocystadenoma papilliferum [3], and other eccrine poromas [8].

## Conclusions

Poroma is a benign adnexal neoplasm arising from intraepidermal cells of eccrine sweat ducts. Although rare, they should be considered in the differential diagnosis of eyelid tumors, especially when malignant. Although it is a benign tumor, wide local excision with free edges should be performed. They have a possible risk of malignant transformation to porocarcinoma or recurrence.
